# Air in the tires: towards an achievable, efficacious and timely diagnosis for Chagas disease

**DOI:** 10.1590/0074-02760210444chgsa

**Published:** 2022-06-01

**Authors:** Andrea Angheben

**Affiliations:** 1 IRCCS Sacro Cuore Hospital

In their recent outstanding paper “Parasitological, serological and molecular diagnosis of acute and chronic Chagas disease: from field to laboratory”,^1^ Schijman and colleagues give a complete overview of the current status of diagnosis of Chagas disease (CD) in a real-life perspective. The topic deserves attention for a complex of reasons:

- the rapid evolution of diagnosis techniques (as a consequence of the globalisation of the disease since the beginning of this century) not timely followed by significant and authoritative change in recommendations/guidelines;

- in the literature, the abundance of diagnostic studies mainly designed in a retrospective way, thus being at risk of accuracy overestimation;

- the lack/paucity of and hard access to biologic reference standards and external quality control programs;

- the paradox of a high underdiagnosis rate across the world (it has been estimated that less of 1% of CD affected patients has access to treatment,^2^ and access to diagnosis is a clear determinant) versus a high number of commercially-available tests;

- the need of evidence-based strategies to apply accurate diagnostic techniques in real life settings such as peripheral/suburban or rural contexts (for instance point-of-care diagnosis of congenital CD).

Since a decade, the World Health Organization (WHO) launched the “tricycle strategy” for the program of control of CD. This iconic picture recalls basic concepts to deal with the burden of CD: a driving wheel represents a surveillance/information system which is needed to keep the right direction, two “carrying” wheels represent adequate case management and transmission control, the driver are the key people involved in the program with the collaboration of social actors.^3^ In this framework, achievable, efficacious and timely diagnosis is the air in the tires.

Too often, access to diagnosis and in a more general breath, diagnostic approaches are left beyond. Research and generation of evidence are not focused on diagnosis. Many issues still remain unanswered:

- the role of natural populations of the parasite, organised in the so-called discrete typing units (DTUs), influence accuracy of tests;

- the difficult application of golden standard diagnostics in field conditions;

- the lack of consensus in screening and diagnostic strategies in different settings and populations;

- the scarcity of methodologically robust studies for the evaluation and comparison of diagnosis tests in the context of a plethora of commercially-available or in-house assays;

- the harmonisation of various approaches to diagnosis, under the guide of the WHO.

Furthermore, according to Alonso-Padilla et al.^4^ and a Delphi study of the Chagas Coalition Working Group (Available form: https://chagas-isglobal.com/wp-content/uploads/2021/04/Pinazo_et_al_Poster_7p.pdf), two other important diagnostic issues remain unanswered: how can we determine a therapeutic failure and the role (and the feasibility of application) of molecular techniques for the assessment of treatment failure.

In the last decades, WHO recommendations set the principle for the application of different tests on the base of the available literature and the opinion of experts, convened to reach a consensus on debated points.

Three documents of reference concerning (also) CD diagnosis are available in this sense: “Control of Chagas disease : second report of the WHO expert committee, (WHO technical report series; 905) published in 2002 (Available from: https://apps.who.int/iris/bitstream/handle/10665/42443/WHO_TRS_905.pdf?sequence=1&isAllowed=y); “WHO Consultation on International Biological Reference Preparations for Chagas Diagnostic Tests WHO, Geneva, 2007” (Available from: https://www.who.int/bloodproducts/ref_materials/WHO_Report_1st_Chagas_BRP_consultation_7-2007_final.pdf) and Pan American Health Organization (PAHO) Guidelines for the Diagnosis and Management of Chagas Disease”, 2020 (Available from: https://iris.paho.org/bitstream/handle/10665.2/49653/9789275120439_eng.pdf?sequence=6&isAllowed=y).

The current recommendation and common strategy for CD diagnosis in the chronic phase is the combination of two serology assays to increase accuracy, one conventional (based on crude antigens or parasite lysate) and one non-conventional (based on recombinant antigens). Although not clearly stated in the official documents, the two tests are intended employed with different techniques. Nevertheless, it is not rare that laboratories across the world use the combination of two enzyme-linked immuno sorbent assays (ELISAs) based on different antigens.

With the appearance on the panorama of new standardised high sensitive and high specific serology assays such as chemiluminescent immunoassays, the need of the strategy of a paired serology has been discussed.^5^ However, if one single but expensive and resource-demanding test could theoretically overcome accuracy limitations of ELISAs, the need of an increase of case-detection can benefit from the use of point-of-care tests or rapid diagnostic tests (RDTs) which could be done also in resource-limited settings (rural regions of low-income countries) or places difficult to reach. Schijman and colleagues present pros and cons of a paired serology diagnosis in chronic CD based on RDTs. If the principal benefit is the feasibility of this approach for field surveys, with an apparently good performance, low cost and time sparing, a limitation to the use of RDTs is the scant evidence on their accuracy, even if paired, on the different DTUs. Most if not almost all prospective studies have been conducted in the Bolivian setting where DTU V is prevalent^6^ but few studies have been currently published on the accuracy of RDTs in regions where DTU I is prevalent.^7^ In these areas, such as Central America and Mexico, RDTs have an unacceptable low sensitivity. In a systematic review and meta-analysis based on prospective studies, the pooled sensitivity of all studied RDTs resulted enough good to recommend them for screening in endemic areas (particularly the South Cone of Latina America) but too low to recommend them as stand-alone tests for case detection in a non-endemic context, as a negative result cannot rule out a *Trypanosoma cruzi* infection with reasonable certainty.^8^

For these reasons, I would be desirable to join efforts to improve RDTs from the technical point of view and on the other hand, to collect solid evidences on the best application of RDTs.

Nevertheless, in the scenario of epidemiological surveys, the PAHO recommends using an ELISA or a RDT; this is referred as a strong recommendation taking into account the moderate degree of certainty on the accuracy of the different interventions but also the need to respect equity and obtain economic advantages.

Congenital transmission of CD accounts of the principal way of transmission in non-endemic countries and represents one of the mayor challenges in endemic countries.^9^ In 2010, the 63rd World Health Assembly urged governments to “establish systems of early detection of cases”, particularly “congenital infections in new-borns”; then, in 2018, the WHO has shifted its objective from control to elimination of congenital CD. The evidences in support of this objective are the high efficacy in preventing congenital transmission of *T. cruzi* (currently 100%) of trypanocidal treatment for women in childbearing age.^9^ Second, treatment of newborns/children with either benznidazole or nifurtimox (the two available trypanocidal medicines) achieve a cure rate close to 100%.

On this base, the first step to control vertical transmission is the screening of mothers or better, according to Carlier et al., of three populations at risk: girls and female adolescents, women in childbearing age and pregnant women.^9^ However, with few exceptions, the majority of countries (mainly in non-endemic area) across the world do not include CD screening as a part of the antenatal care.

Currently, it is mandatory to include *T. cruzi* testing into screening programs before/during pregnancy. Vertical interventions do not represent a sustainable approach and have already exhausted their task having demonstrated the cost-effectiveness of the screening of all at risk women.^10^ A paradigm change is needed and CD should be incorporated as a part of perinatal care algorithms.

In this sense, the acronym ToRCH (toxoplasmosis, rubella, cytomegalovirus and herpes) which represent the complex of common agents of infection which can pose at risk a healthy birth, could be modified to ToRCH^2^ (ToRCH square) where CH represents also Chagas disease.

Moreover, the offspring of affected women should go through simplified diagnostic algorithms, thus reducing the risk of loss to follow-up. The role of molecular biology techniques, although promising, has not been clearly established and classical sequential microscopy/serology approach is not universally reliable, particularly in non-endemic countries. Again, it is necessary to implement sustainable programs of diagnosis in the perinatal period, adapted to the requirements of the site and health systems. Point-of-care assays are urgently expected.

In conclusion, I add my voice to that of Schijman and colleagues asking the scientific community to focus on generating robust evidences through high quality studies and to fill the above-mentioned diagnostic gaps. On the other hand, national and international institutions should take charge of reordering the available evidence, simplifying diagnostic pathways and valorising the available tools according to the settings of application. More than one century after its first description, it is time to shift from a vertical to a horizontal approach, incorporating CD into primary health care and preventive medicine of national health systems.

Comments on the article: Schijman AG, Alonso-Padilla J, Longhi SA, Picado A. Parasitological, serological and molecular diagnosis of acute and chronic Chagas disease: from field to laboratory. Mem Inst Oswaldo Cruz. 2022; 117: e200444.

## References

[1] Schijman AG, Alonso-Padilla J, Longhi SA, Picado A (2021). Parasitological, serological and molecular diagnosis of acute and chronic Chagas disease from field to laboratory. Mem Inst Oswaldo Cruz.

[2] Ribeiro I, Sevcsik AM, Alves F, Diap G, Don R, Harhay MO (2009). New, improved treatments for Chagas disease from the R&D pipeline to the patients. PLoS Negl Trop Dis.

[3] Sanmartino M, Forsyth CJ, Avaria A, Velarde-Rodriguez M, Gómez i Prat J.Albajar-Viñas P (2021). The multidimensional comprehension of Chagas disease Contributions, approaches, challenges and opportunities from and beyond the Information, Education and Communication field. Mem Inst Oswaldo Cruz.

[4] Alonso-Padilla J, Abril M, Alarcon de Noya B.Almeida IC.Angheben A.Araujo Jorge T (2020). Target product profile for a test for the early assessment of treatment efficacy in Chagas disease patients an expert consensus. PLoS Negl Trop Dis.

[5] Abras A, Gallego M, Llovet T, Tebar S, Herrero M, Berenguer P (2016). Serological diagnosis of chronic Chagas disease Is it time for a Change?. J Clin Microbiol.

[6] Abras A, Gallego M, Munoz C, Juiz NA, Ramirez JC, Cura CI (2017). Identification of Trypanosoma cruzi discrete typing units (DTUs) in Latin-American migrants in Barcelona (Spain). Parasitol Int.

[7] Sosa-Estani S, Gamboa-Leon MR, Del Cid-Lemus J, Althabe F, Alger J, Almendares O (2008). Use of a rapid test on umbilical cord blood to screen for Trypanosoma cruzi infection in pregnant women in Argentina, Bolivia, Honduras, and Mexico. Am J Trop Med Hyg.

[8] Angheben A, Buonfrate D, Cruciani M, Jackson Y, Alonso-Padilla J, Gascon J (2019). Rapid immunochromatographic tests for the diagnosis of chronic Chagas disease in at-risk populations a systematic review and meta-analysis. PLoS Negl Trop Dis.

[9] Carlier Y, Altcheh J, Angheben A, Freilij H, Luquetti AO, Schijman AG (2019). Congenital Chagas disease updated recommendations for prevention, diagnosis, treatment, and follow-up of newborns and siblings, girls, women of childbearing age, and pregnant women. PLoS Negl Trop Dis.

[10] Sicuri E, Munoz J, Pinazo MJ, Posada E, Sanchez J, Alonso PL (2011). Economic evaluation of Chagas disease screening of pregnant Latin American women and of their infants in a non-endemic area. Acta Trop.

